# ABO Blood Groups Do Not Predict *Schistosoma mansoni* Infection Profiles in Highly Endemic Villages of Uganda

**DOI:** 10.3390/microorganisms9122448

**Published:** 2021-11-27

**Authors:** Rachel Francoeur, Alon Atuhaire, Moses Arinaitwe, Moses Adriko, Diana Ajambo, Andrina Nankasi, Simon A. Babayan, Poppy H. L. Lamberton

**Affiliations:** 1 Institute of Biodiversity, Animal Health and Comparative Medicine, University of Glasgow, Glasgow G12 8QQ, UK; simon.babayan@glasgow.ac.uk; 2 Welcome Centre for Integrative Parasitology, University of Glasgow, Glasgow G12 8QQ, UK; 3 Faculty of Science and Engineering, Department of Biological Sciences, University of Chester, Chester CH1 4BJ, UK; 4 Vector Control Division, Ministry of Health, Kampala P.O. Box 1661, Uganda; aaronatuhaire@gmail.com (A.A.); moses0772359814@gmail.com (M.A.); adrikomoses@gmail.com (M.A.); n1andrina@gmail.com (A.N.)

**Keywords:** schistosomiasis, blood group, susceptibility, rhesus, coinfection, STH

## Abstract

*Schistosoma mansoni* is a parasite which causes significant public-health issues, with over 240 million people infected globally. In Uganda alone, approximately 11.6 million people are affected. Despite over a decade of mass drug administration in this country, hyper-endemic hotspots persist, and individuals who are repeatedly heavily and rapidly reinfected are observed. Human blood-type antigens are known to play a role in the risk of infection for a variety of diseases, due to cross-reactivity between host antibodies and pathogenic antigens. There have been conflicting results on the effect of blood type on schistosomiasis infection and pathology. Moreover, the effect of blood type as a potential intrinsic host factor on *S. mansoni* prevalence, intensity, clearance, and reinfection dynamics and on co-infection risk remains unknown. Therefore, the epidemiological link between host blood type and *S. mansoni* infection dynamics was assessed in three hyper-endemic communities in Uganda. Longitudinal data incorporating repeated pretreatment *S. mansoni* infection intensities and clearance rates were used to analyse associations between blood groups in school-aged children. Soil-transmitted helminth coinfection status and biometric parameters were incorporated in a generalised linear mixed regression model including age, gender, and body mass index (BMI), which have previously been established as significant factors influencing the prevalence and intensity of schistosomiasis. The analysis revealed no associations between blood type and *S. mansoni* prevalence, infection intensity, clearance, reinfection, or coinfection. Variations in infection profiles were significantly different between the villages, and egg burden significantly decreased with age. While blood type has proven to be a predictor of several diseases, the data collected in this study indicate that it does not play a significant role in *S. mansoni* infection burdens in these high-endemicity communities.

## 1. Introduction

Schistosomiasis is a debilitating, neglected tropical disease with over 240 million people infected globally [1]. Despite over a decade of mass drug administration (MDA) in many sub-Saharan African countries, hyperendemic hotspots persist, where individuals are repeatedly heavily and rapidly reinfected [2,3]. A range of factors may contribute to persistent hotspots relating to the environment, such as sanitation or snail habitat suitability [4,5]; and to parasite or host influences, including variation in genetic background, immune responses [6], and/or drug efficacy [7]. Individuals can vary in their predisposition to helminth infections [8], and, in particular, blood group has been hypothesised to contribute to schistosome susceptibility [9].

Associations between blood type and disease susceptibility or severity have been described [10]. A and/or B antigens are thought to be advantageous in conferring resistance to some pathogens [11]. For example, the prevalence of *Ancylostoma duodenale* (Hookworm) was found to be higher in individuals with an O blood type than in those with A or B antigens [12]. However, in some instances, A and/or B antigens increase susceptibility to some diseases. For example, non-O groups have been associated with increased risk and severity of acute respiratory syndrome coronavirus 2 (SARS-CoV-2) infection [13]. Individuals with blood group O appear to have a lower susceptibility to the virus [14], and group A shows a higher susceptibility to infection [15]. Susceptibility to both malaria and tuberculosis is highest in individuals with blood group B, due to the structurally similar α-Gal surface proteins on blood-cell and pathogen surfaces [16]. Variance in disease susceptibility amongst the blood types is thought to be mediated by cross-reacting responses, where anti-A or anti-B antibodies detect foreign proteins expressing A and B motifs or conversely do not detect them if they do not possess these antibodies [10,17]. Pathogens expressing A- and/or B-type epitope sequences are more likely to be detected by the host immune system of individuals with an O-type blood group [18]. Geographical variation among blood-type groups illustrates that pathogen selection can influence co-evolution between blood-group antigens and pathogens [19].

There are few studies solely investigating the impact of Rhesus (Rh) blood groups on disease. Variation in surface proteins on blood cells in Rh-negative individuals have been found to facilitate viral binding in West Nile Virus and thereby increase susceptibility of contracting West Nile Virus [20]. A recent study has found a correlation between Rh positivity and a predisposition to SARS-CoV-2 [21]. The risk of contracting dengue haemorrhagic fever is increased in Rh-positive individuals [22]. These studies suggest that there is a correlation between Rh blood types and infectious disease, but the effect is disease-specific.

One mechanism parasites use to evade immune defence is through the adhesion to host proteins. Several studies have reported a relationship between antigen acquisition and evasion of host immune responses [9]. Early work in this area has described the occurrence of adsorption of host antigens containing glycoproteins and glycolipids by schistosomes. This is also thought to include blood groups [23,24,25]. Adhesion of host proteins is understood to occur within the membranocalyx or inter-membrane zones of the schistosome tegument, wherein studies have detected internalised host-derived immunological proteins [26,27]. Further, structural similarities occur between human-blood proteins in A blood groups (GalNAc) and schistosome polysaccharides [28,29], potentially leading to increased susceptibility to schistosome infections and greater infection intensities. Meanwhile, individuals with B or O blood types may be more efficient at eliciting an anti-A immune response [29]. Therefore, the occurrence of blood-group protein adhesion by schistosomes may contribute to the magnitude of immunological detection by the host and might explain variation in prevalence, intensity, and severity among endemic populations.

Of the few studies that have attempted to establish a relationship between blood group and prevalence, intensity or severity of *S. mansoni* infections, the results have been conflicting [9]. A recent study found that individuals with an AB blood type are four times more likely to have a high egg burden than those with an O group [30]. This study focused on infection intensity based on one stool sample and did not look at the impact of blood group on clearance after treatment. Several studies have found that patients with A blood groups display an increased schistosomiasis disease severity, and O groups exhibit the lowest prevalence and intensity of schistosomes [31,32,33]. These studies also focused on infection status at the time of sampling and did not consider the longitudinal impact of blood type on schistosome infection dynamics over time or with drug treatment, and subsequent clearance, and reinfection. One study that did look at longitudinal data assessed the annual incidence of schistosome infection and found that individuals with A-type blood had the highest intensities and annual incidence of both *S. mansoni* and *S. haematobium* [34]. Conversely, another study found that the group with most prevalent *S. mansoni* infections at a single time point was type B [35]. Earlier work conducted by Katz et al. found no significant difference between blood groups and *S. mansoni* prevalence amonst patients with hepatosplenic schistosomiasis [36]. A study by Kassim and Eiezie (1982) examined the rate of *Schistosoma haematobium* and malaria infection and found that the frequencies of blood groups were not associated with infection rates [37]. However, diagnostic methods and sensitivities, and host–pathogen interactions, including the potential effect of host blood type, differ between *S. mansoni* and *S. haematobium* [38]. The above discrepancies indicate a need to expand knowledge on the interaction between blood type and *S. mansoni* infection dynamics in an endemic setting [9]. There are currently no published studies looking at the role blood type plays on treatment efficacy in schistosome infections or any longitudinal studies to determine whether blood type is a risk factor for repeated or rapid re/infection. Furthermore, no studies have investigated the effect host blood group may have on coinfection with soil-transmitted helminths (STHs) and schistosomes, despite how they commonly co-occur and are often co-treated at the same time through many national control programmes.

The purpose of this study was to evaluate the association between host blood type and its effect on *S. mansoni* infection dynamics, with repeated observations over time in school-aged children (SAC). Specifically, the aim was to analyse repeat monitoring data about infection/coinfection predisposition, prevalence, intensity, clearance, and reinfection post-treatment, so that a comprehensive representation could be assessed in relation to the impact that blood group may have on these parameters in areas where these *S. mansoni* dynamics remain high despite over 15 years of MDA.

## 2. Materials and Methods

A total of 630 school children between the ages of 6 and 14 were recruited from three primary schools in Mayuge district Uganda, all in high *S. mansoni* endemicity villages along the shores of Lake Victoria. An equal ratio of boys and girls were recruited and evenly distributed between year age groups. Two hundred and seventy children were recruited from Bugoto Lake View Primary School (0° 1924.09′ N, 33°3741.40′ E) in September 2017. One hundred and eighty children were recruited from both Bwondha Primary School (0°117.15′ N, 33°3339.87′ E) and Musubi Church of Uganda God Primary School (0°1849.45′ N, 33°3947.50′ E) in September 2017 and October 2017, respectively.

**Figure 1:**
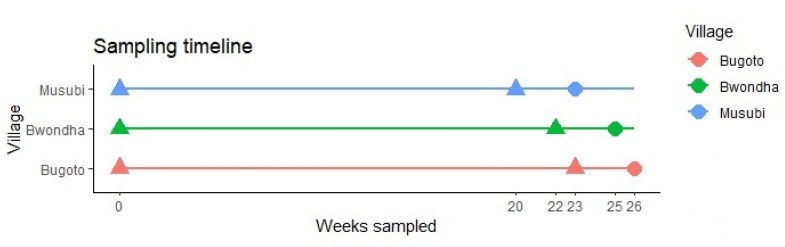
Kato–Katz thick smear sampling (three days of duplicate Kato–Katz at each time point) and praziquantel treatment timeline, indicating the weeks sampling occurred in each school. Triangles indicate praziquantel administration given, after stool samples were collected, on that week, and circles denote treatment was only administered to children with *Schistosoma mansoni* detected infections.

In each school, three days of duplicate Kato–Katz thick smears were prepared, in accordance with established protocol [39], from three stool samples provided by participants. On the third day, after sample collection, the participants were all given a meal of porridge and butter an hour prior to observed treatments with 40 mg/kg of praziquantel and 400 mg of albendazole. Individuals from the three schools were all followed up approximately six months later to quantify reinfection rates; they were then retreated with 40 mg/kg praziquantel and followed up with three weeks later to quantify clearance post-treatment. Sample and treatment dates are shown in Figure 1.

In September 2019, participants provided a finger-prick blood sample to identify blood type. A small drop of blood was spotted onto glass slides and mixed with anti-A, anti-B monoclonal sera and an anti-D blend (Alpha Laboratories, Ltd., Hampshire, UK). The slides were checked for agglutination by trained technicians within 10 min of mixing blood and antisera.

Criteria for inclusion in the blood group study included parental consent, child assent, and the provision of a finger-prick blood sample and a stool sample at one or more time point/s.

Because the schools were not all sampled on the same dates, the number of weeks at follow-up was controlled for in the analysis. Participants were excluded from the study if weight and height information collected at baseline or blood-group data were missing. Loss to follow-up was high; therefore, only 265 individuals were included in the final analysis.

To assess the role that blood type plays in predisposition to infection and to determine its effect on clearance, prevalence, and intensity, a generalised linear mixed-effects model was performed, using the glmmTMB [40] package in R ver. 3.5.1 [41]. Negative binomial model assumptions were assessed by using several error structures, including both nbinom1 and nbinom2 parameters, as well as Poisson distributions [40]. The nbinom2 error distribution had minimal residual variance compared with the other error structures assessed and was therefore chosen as the best fit for the data. Residuals were assessed for uniformity, using the DHARMa package [42]. Briefly, a bootstrapped simulation of residuals was performed, along with Kolmogorov–Smirnov uniformity tests; quantile regression of residuals versus fitted model values and Q–Q plots of expected versus observed residual values were computed in order to validate the model.

The initial model included the age of the participants (6–14 years), status of co-infection with STH, gender, and BMI values as fixed effects, because these parameters are variables known to affect schistosome infection intensity and prevalence. The time point at which Kato–Katz samples were obtained and the school in which the child attends were also included in the model to assess the effect of blood type on *S. mansoni* intensity, clearance, and variability between the schools.

Model selection was performed by using the drop1 function in R, which compares all possible models that can be constructed by each time, removing the lowest estimate variable, which included the removal of BMI, gender, and Rh type, to obtain a parsimonious final model. ABO blood group was not a significant predictor, but because of its biological importance, it was retained during model selection.

## 3. Results

**Figure 2:**
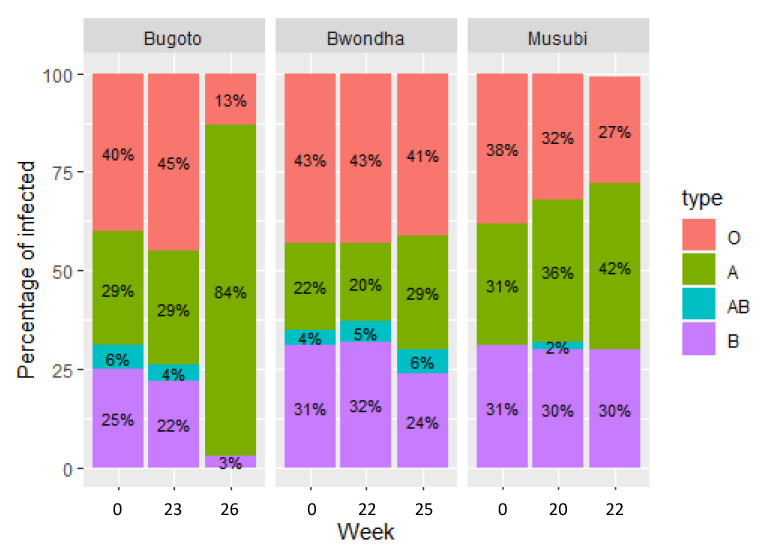
*Schistosoma mansoni* infection prevalence, split by school, host blood type, pretreatment, six months post-treatment, and then three weeks later after re-treatment. Blood groups are denoted by colour.

In the Ugandan villages of Bugoto, Bwondha, and Musubi, which have experienced over 15 years of regular praziquantel administration (either annually or bi-annually), *S. mansoni* infection was detected in 190 (72%) individuals (*n* = 265) across the schools, with prevalences of 86% in Bugoto, 71% in Bwondha, and 57% in Musubi and mean infection intensities of 164.6 EPG (95% CI: 113.9–215.3), 108.4 EPG (95% CI: 73.4–143.5), and 92.6 EPG (95% CI: 53.6–131.7), respectively, at baseline. The most commonly occurring blood group was O, with 39% of individuals in Bugoto, 49% in Bwondha, and 39% in Musubi. There was no significant difference between proportion of blood groups observed between the schools (*p* = 0.390). Type AB occurred significantly less often (*p* < 0.001) than all other groups, with a prevalence of 6%, 3%, and 1% in Bugoto, Bwondha, and Musubi, respectively. Only 4.8% of individuals from all three schools had an Rh- blood group, and no individuals were Rh- in Musubi. There was no significant association between blood groups and *S. mansoni* prevalence (*p* = 0.811). However, significant variation in prevalence occurred between the schools at each time point (*p* < 0.001) (Figure 2). 

**Figure 3:**
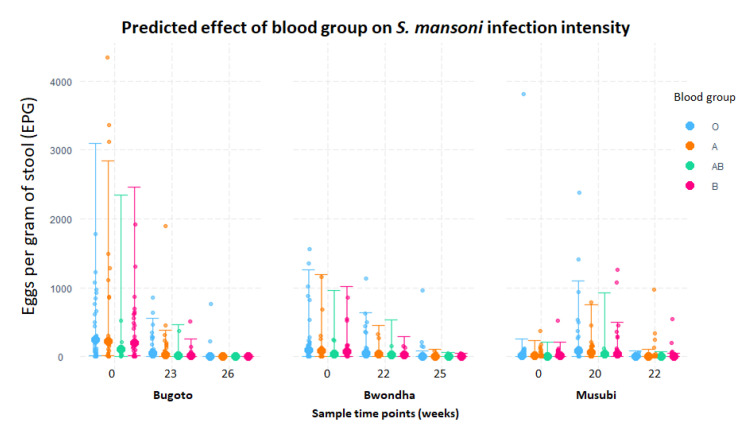
Relationship between blood group and infection intensity (eggs per gram of stool (EPG)) categorised by village school. The colour-coded effect lines underlaid by raw data points illustrate the model predictions that there is no significant association between blood group and EPG.

Infection intensity varied between the schools at baseline and each follow-up (*p* < 0.001), but there was no association between blood group and infection intensity (*p* = 0.818) (Figure 3). 

The incidence of Rh- individuals was low, at 4.8%, but slightly higher than other studies conducted in similar regions [43,44]. However, because of its low estimate value, Rh was removed from the final model.

Body mass index (BMI) was calculated based on the World Health Organization’s (WHO) growth standards [45]; however, the best-fit model did not include this parameter, due to the low estimate of this variable.

At baseline, 27 (10%) individuals across all three schools had a STH coinfection, while only three (1%) were mono-infected with STHs. There was no significant relationship between blood group and mono or coinfection with STHs (*p* = 0.101).

## 4. Discussion

The Ugandan Ministry of Health has been conducting community-wide MDAs to control schistosomiasis in the Mayuge district since 2004. As with many areas in sub-Saharan Africa, despite these efforts, the burden remains high. This may be partly explained by low MDA coverage [46], but even when children are given observed praziquantel treatment, clearance rates are low [47], many are very rapidly reinfected [2,47], and several remain heavily infected over repeated time points. This could indicate a behavioural or a biological predisposition to rapid and heavy reinfections. This investigation, part of a larger host-factor study, focused on blood group as a potential biological factor which may be driving the high *S. mansoni* prevalence, intensities, low clearance, and rapid reinfections seen in this area of Uganda. However, no association was found between host blood-group type and *S. mansoni* infection prevalence or intensity at baseline or over time with two praziquantel treatments, and there was no association with clearance rates, egg reduction rates or co-infection with STHs.

The examination of the impact of blood type on disease incidence is important to help further our understanding of host factors driving the persistence of infection transmission. Research looking specifically at *S. mansoni* has produced contradictory results regarding the influence that blood type has on schistosomiasis. The results of this study showed no correlation between blood type and *S. mansoni* infection dynamics: prevalence, intensity, clearance, or reinfection rates. In the majority of other publications, as outlined above, schistosomiasis infection status was also determined using the Kato–Katz method; however, most of the other Kato–Katz/blood-type studies only obtained one stool sample at one time point. A more robust depiction of the infection status of the cohort is provided by using duplicate Kato–Katz slides over three days and repeating at follow-up time points [48,49], as was conducted in this study. Obtaining only one slide per time point increases the risk of false negatives [50]. Berhe et al. noted that false negatives occurred when using either a single Kato–Katz reading or three slides at a rate of 68% and 21%, respectively [51]. To mitigate limitations of the Kato–Katz method, the participants in this study provided stool samples at three time points, for a total of 18 (six per time point) Kato–Katz slides, thus enabling a better depiction of infection status and changes exhibited over time. Expanding on previous studies by assessing the effect of blood group longitudinally, this fully powered study indicates that it does not play a role in the risk of reinfection and clearance rates in these highly endemic villages in Uganda. It may be that the force of infection is so high in these villages that individuals become infected despite the presence or absence of blood-group antigens. However, there was also no association between blood group and infection intensities over time, which is a more sensitive measure for a predisposition to infections in such high force of infection foci and would be expected to demonstrate an effect if it existed here.

The incidence of Rh- individuals at 4.8% is slightly higher than other studies conducted in similar regions [43,44]. However, its role in *S. mansoni* infection dynamics could not be determined in this study, due to the disproportionate number of individuals with Rh- blood.

The association between age and infection prevalence and intensity suggests that the risk of infection decreases with age. A relationship between age and infection intensity has been described in numerous studies examining schistosomiasis and is an important risk factor for infection [52,53,54,55]. The driving factor behind this occurrence is thought to be due to repeated exposure to *S. mansoni* causing an increased immunity toward the pathogen [53,56]. It has also been suggested that an increase in age relates to changes in water contact behaviour, thereby reducing the opportunity for reinfection [55,57]. The association with age would suggest that continued work investigating age-related immunity may be imperative to advancing current knowledge to improve treatment and prevention measures.

The relationship between the school an individual attended and *S. mansoni* infection dynamics had a significant impact on infection profile. Several factors may be driving this intra-divergence in infection dynamics, including variances in MDA history, MDA coverage, water-contact behaviour, accessibility to sanitation and/or safe water, parasite or host genetics, and host immunity. While blood group is not associated with *S. mansoni* infection dynamics in this hyper-endemic region, further work is required to assess the influence of other within-host factors that may play a role in the risk of *S. mansoni* infection, in order to develop more targeted control programmes.

## Data Availability

Data are available in a publicly accessible repository. The data presented in this study are openly available in Enlighten: Research Data at [http://dx.doi.org/10.5525/gla.researchdata.1219], reference number [1219].
